# *Elsholtzia ciliata* (Thunb.) Hyl. Extracts from Different Plant Parts: Phenolic Composition, Antioxidant, and Anti-Inflammatory Activities

**DOI:** 10.3390/molecules25051153

**Published:** 2020-03-05

**Authors:** Lauryna Pudziuvelyte, Mindaugas Liaudanskas, Aiste Jekabsone, Ilona Sadauskiene, Jurga Bernatoniene

**Affiliations:** 1 Department of Drug Technology and Social Pharmacy, Lithuanian University of Health Sciences, Eiveniu 4, LT-50161 Kaunas, Lithuania; lauryna.pudziuvelyte@lsmuni.lt; 2 Laboratory of Pharmaceutical Sciences, Institute of Pharmaceutical Technologies, Lithuanian University of Health Sciences, Sukileliu ave. 13, LT-50162 Kaunas, Lithuania; mindaugas.liaudanskas@lsmuni.lt (M.L.); aiste.jekabsone@lsmuni.lt (A.J.); 3 Department of Pharmacognosy, Lithuanian University of Health Sciences, Eiveniu str. 4, LT-50161 Kaunas, Lithuania; 4 Laboratory of Molecular Neurobiology, Neuroscience Institute, Lithuanian University of Health Sciences, Eiveniu str. 4, LT-50161 Kaunas, Lithuania; ilona.sadauskiene@lsmuni.lt

**Keywords:** *Elsholtzia ciliata*, anti-inflammatory activity, natural compounds, polyphenols, antioxidant, HPLC–ABTS post-column, rosmarinic acid

## Abstract

Polyphenols play an important role on the health-promoting properties of humans. Plants belonging to *Lamiaceae* family are known as rich source of phenolic compounds. The current work aimed to evaluate the phenolic compounds, antioxidant, and anti-inflammatory activity of *Elsholtzia ciliata* (Thunb.) Hyl. ethanolic extracts from leaf, stem, flower, and whole herb. Twelve compounds were identified in ethanolic extracts using high-performance liquid chromatography (HPLC). The HPLC analysis revealed that chlorogenic acid, rosmarinic acid, and rutin were predominant compounds in ethanolicic extracts. Using HPLC-ABTS (2,2’-azino-bis(3-ethylbenzothiazoline-6-sulfonic acid)) post-column assay, avicularin, chlorogenic, and rosmarinic acids were identified as the predominant radical scavengers in all ethanolic extracts. All tested preparations significantly reduced the level of secretion of proinflammatory cytokines TNF-α, IL-6, and prostaglandin E2 induced by lipopolysaccharide treatment in mouse peritoneal macrophage cell culture. Stem and flower extracts were most efficient in reducing cytokine release, but leaf extract demonstrated stronger effect on prostaglandin E2 secretion. This is the first study exploring antioxidant efficiency by HPLC–ABTS post-column method and investigating anti-inflammatory activity of ethanolic extracts from *E. ciliata* different plant parts.

## 1. Introduction

Natural substances and medicinal herbs have been traditionally administered to treat or prevent various diseases all over the world. A number of aromatic, spicy, and medicinal plants accumulate various organic active compounds, which can be classified into four major classes: Phenolic compounds, alkaloids, terpenoids, and sulfur-containing compounds [1,2]. These active compounds differ in their physical and chemical properties, structures, and action mechanisms. Phenolic compounds, or polyphenols, make the largest group of phytochemicals with great chemical diversity—more than 8000 structural variants [3,4]. Phenolic compounds are characterized by the presence of one or more aromatic rings bearing one or more hydroxyl moieties. The structure of these phytochemicals varies from simple molecules, such as phenolic acids, to highly polymerized molecules, such as condensed tannins. The main polyphenol subgroups are defined into these groups: Flavonoids, phenolic acids, lignans, tannins, and stilbenes [3,5,6,7,8]. The most commonly occurring polyphenols are phenolic acids and flavonoids [9]. In herbs, polyphenols ensure protection against UV light, oxygen and nitrogen species, pathogens, and parasites [9,10,11,12].

Animal, human, and epidemiologic studies have concluded that beneficial effects of polyphenols are frequently related to their antioxidant activity, that might have preventive or therapeutic effects for neurodegenerative disorders, cardiovascular diseases, cancer, osteoporosis, pancreatitis, gastrointestinal problems, lung damage, and obesity [2,3,4,9,13,14]. One of the most common condition in disease is inflammation characterized by increased oxidative stress and release of specific cytokines and mediators promoting inflammatory activation. Both oxidative bursts can be induced by the inflammatory cytokines as well as release of the cytokines can be caused by increased oxidation level [15]. Thus, phytocompounds with antioxidant activity are also expected to possess anti-inflammatory properties.

*Elsholtzia ciliata* (Thunb.) Hyl.—an annual plant belonging to *Lamiaceae* Martinov family—is widely distributed throughout China, Korea, and Europe [16]. The mint family (*Lamiaceae*) is an important medicinal flowering plant family that contains about 236 genera and more than 6000 species [17]. As usual in plants, phenols belong to the largest group of secondary metabolites in *Lamiaceae* family, and they exhibit multidirectional biological activity [18]. The crude extract from *E. ciliata* contains not only phenols, but also essential oil, flavonoids, steroids, and triterpenes [16]. In traditional medicine, *E. ciliata* has been used for the treatment of headache, fever, diarrhea, edema, blood clotting, gastralgia, dysphonia, nephritis, and throat infections [16,19,20]. According to scientific literature, *E. ciliata* is a valuable bioactive source of natural antioxidants [21]. Extracts of *E. ciliata* possess anti-inflammatory [21], antiviral, antibacterial, antioxidant [22], anticancer [23], and vasorelaxant [24] effects.

Spectrophotometric assay is the most popular method for determination of total antioxidant potency of various plant materials [25]. However, in the past few years, more advanced online HPLC post-column assay has been developed and applied successfully for rapid screening and identification of antioxidants from the crude extracts of medicinal herbs [26]. Model oxidation systems of DPPH (2,2-diphenyl-1-picrylhydrazyl), FRAP (Ferric Reducing Antioxidant Power), and ABTS (2,2’-azino-bis(3-ethylbenzothiazoline-6-sulfonic acid))radicals are highly effective, rapid, and possess other important advantages compared with conventional strategies for identification of antioxidants from complex mixtures [27]. There are a lot of studies that have determined antioxidant activity of essential oils, phenolic compounds, and others, but only a few researches have been published about the antioxidant activity of individual compounds [27,28]. *E. ciliata* is not an exception—there is only one publication from Liu et al. describing the antioxidant effect of aqueous extracts from different parts of *E. ciliata* [29]. In our previous study, we have determined the chemical composition of phenolics in ethanolic extracts produced from dried whole herb [24]. However, there is no information available about the chemical composition of phenolic compounds and antioxidant activity, neither anti-inflammatory properties of different parts of ethanolic extracts from different parts of *E. ciliata*. Therefore, the objective of this study was to determine the main phenolic compounds in the ethanolic extracts of *E. ciliata* leaf, stem, flower, and whole herb. The total phenolics content (TPC) and total flavonoids content (TFC) as well as antioxidant activity in the ethanolic extracts were determined using spectrophotometrical analysis. The antioxidant active compounds (radical-scavenging activity) were identified using online assay with HPLC post-column reactions. Anti-inflammatory properties were evaluated by measuring amounts of inflammatory mediators released from murine peritoneal macrophages stimulated with bacterial lipopolysaccharide in the presence of ethanolic extracts from different parts of the *E. ciliata* plant.

## 2. Results and Discussion

### 2.1. Determination of Total Phenolic Content

**Figure 1:**
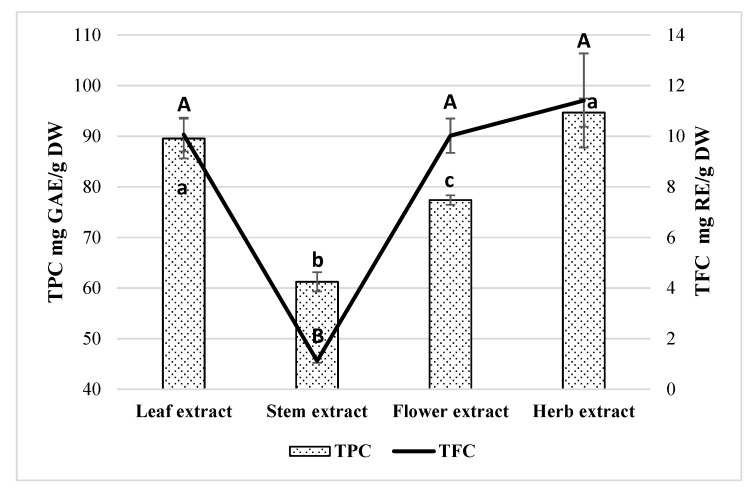
Total phenolic compounds (TPC) and total flavonoid content (TFC) of ethanolic *E. ciliata* extracts. Results are means ± SD. Values with different capital (TFC) and lowercase (TPC) letter(s) are significantly different (*p* < 0.05) measured by Tukey‘s test.

Plant heterogeneity is very widespread and is used for the selection of garden and medicinal plants and for the evaluation of the quality of medicinal plant raw materials. Many medicinal plant species are characterized by inter-species chemical diversity, of which the study and evaluation is very important. Chemical diversity studies demonstrate the qualitative and quantitative composition of the active substance within species, varieties, parts of plants, and between different plants of the same species. In order to determine the patterns of the accumulation of biologically active compounds in plants, it is important to identify their composition and content in separate plant organs. Pilot studies were carried out to determine the total content of phenolic compounds and total flavonoids by UV-visible light spectrophotometry in order to evaluate the diversity of the composition of phenolic compounds of different *E. ciliata* plant parts. Studies showed that TPC ranged from 61.25 ± 1.91 to 94.67 ± 1.91 mg gallic acid equivalent (GAE)/g dry weight (DW) (Figure 1).

The amount of phenolics is statistically significantly higher in whole herb (94.67 ± 1.91 mg GAE/g DW), leaf (89.55 ± 3.91 mg GAE/g DW), and flower (77.39 ± 0.94 mg GAE/g DW) extracts than stem (61.25 ± 1.91 mg GAE/g DW) extract (*p* < 0.05). According to Liu et al. [29], the amount of the TPC was significantly higher in root fraction (497.2 ± 24.9 mg GAE/g) and the lowest in stem (213.1 ± 6.2 mg GAE/g) and inflorescence (198.2 ± 10.1 mg GAE/g) fractions of *E. ciliata*. A recent study investigating the extracts of mint, parsley, and coriander obtained from leaf and stem, reported highest amounts of TPC from extracts obtained from leaf (extract of mint (*Lamiaceae* family) leaf—1.24 mg GAE/100 mL, extract of parsley (*Apiaceae* Lindl. family) leaf—1.22 mg GAE/100 mL, extract of coriander (*Apiaceae* family) leaf—1.12 mg GAE/100 mL) [30]. These results showed that the higher value of TPC was obtained from leaf extract than from stem extract. In other study, TPC ranged from 1.33–5.01 g GAE/100 g dry weight—leaf and flower extracts obtained with methanolic or water had higher amounts of phenolic compounds than stem extract produced from *Justica spicigera* Schltdl. (*Acanthaceae* Juss. family) [31]. Researchers in other study evaluated the TPC or TFC of 20 different medicinal plants parts: leaf, stem, and flower [32]. Results showed that TPC ranged from 65 to 500 mg/g^−1^ DM in leaf, 25 to 210 mg/g^−1^ DM in stem, and 95 to 450 mg/g^−1^ DM in flower. It was noticed that amounts of TPC in *Lamiaceae* and *Rosaceae* Juss. species were higher than in other families. These results of the studies mentioned showed the same tendency as in our study that extracts obtained from leaf and flower have higher amounts of phenolic compounds than extracts obtained from the stem. The difference may be due to the different composition and amounts of the phenolic compound in different parts of the plant. The differences may also be due to the extraction solvent used for the extraction, the conditions of extraction, and the quality of the plant material itself (under the conditions of the environment during the growing period, at which stage the raw material was cut, etc.).

### 2.2. Determination of Total Flavonoid Content

The content of flavonoids obtained in the *E. ciliata* extracts is shown in Figure 1. The TFC ranged from 5.06 ± 0.08 to 15.43 ± 1.86 mg RE/g DW. The highest flavonoid levels have been obtained in whole herb (15.43 ± 1.86 mg RE/g DW), flower (14.22 ± 0.67 mg RE/g DW), and leaf (14.16 ± 0.65 mg RE/g DW) extracts. The extract from the stem showed a significantly lower amount of TFC (5.06 ± 0.08 mg RE/g DW) than leaf, flower, and whole herb extracts (*p* < 0.05). Other researchers investigating methanolic extracts of *E. ciliata* plant parts have found TFC between 0.18 and 1.30 g catechin equivalents/100 g dry weight and the order for methanolic extracts was leaf > flower > stem, whereas for the aqueous extracts, this sequence was stem > flower > leaf [31]. In another study, TFC ranged from 1.3 to 3.4 mol/g^−1^ DM in leaf, from 0.8 to 2.9 mol/g^−1^ DM in stem, and from 1.1 to 3.3 mol/g^−1^ DM in flower [32]. The higher value of flavonoids was found in samples of apple leaves belonging to the family of *Rosaceae* Juss., which ranged from 21.59 ± 0.52 to 45.02 ± 0.90 mg RE/g DW [33].

### 2.3. Measurements of Antioxidant Activity in Extracts

After studying the total phenolics and flavonoids content of E. ciliata, it is important to examine and assess an antioxidant activity of different plant parts. The results obtained during studies will be useful to provide a consumer with products rich in antioxidants, will be useful for the assessment and standardization of quality of plant raw materials and their products, and will allow predicting an antioxidant effect of E. ciliata extracts from different plant parts in vivo.

**Figure 2:**
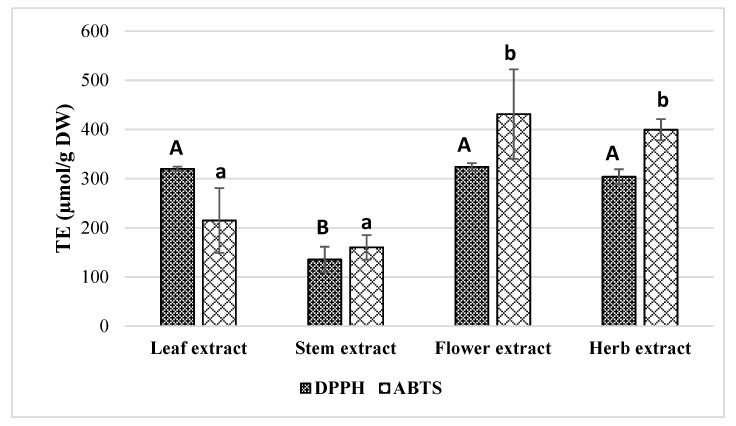
Antiradical (DPPH and ABTS radical scavenging) activities of ethanolic *E. ciliata* extracts. Results are means ± SD. Values with different capital (DPPH) and lowercase (ABTS) letter(s) are significantly different (*p* < 0.05) measured by Tukey‘s test.

**Figure 3:**
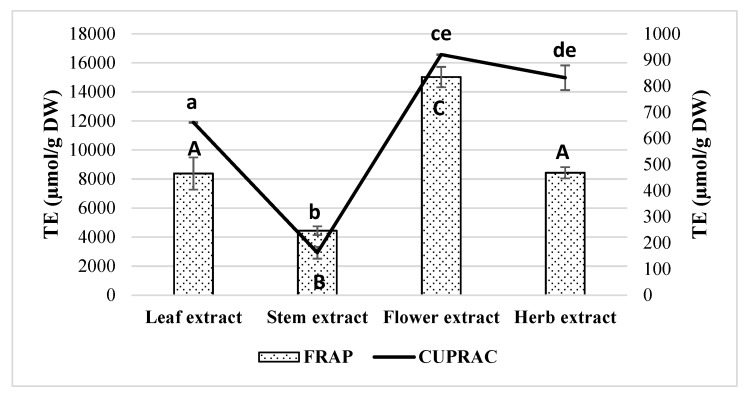
Antioxidant (FRAP and CUPRAC) activities of ethanolic *E. ciliata* extracts. Results are means ± SD. Values with different capital (FRAP) and lowercase (CUPRAC) letter(s) are significantly different (*p* < 0.05) measured by Tukey‘s test.

Herbal extracts contain large amounts of biologically active compounds that have antioxidant activity that gets in the mechanism of the different reactions, so antioxidant effects cannot be analyzed using only one method [34,35]. For these reasons, it is suggested to use at least two or more different methods to determine the antioxidant activity of herbal extracts. To evaluate the antioxidant activity of *E. ciliata* ethanolic extracts, different antioxidant capacity assays (DPPH, ABTS, FRAP, and CUPRAC (Cupric Reducing Antioxidant Capacity)) were employed. The results of antioxidant activity obtained in the ethanolic extracts of leaf, flower, stem, and the whole herb are summarized in Figure 2 and Figure 3.

Overall, stems extract was the sample that revealed the lowest antioxidant potential exhibiting TE values of 135.60 ± 25.89, 160.17 ± 24.89, 4438 ± 304.87, and 16.40 ± 22.80 µmol/g for DPPH, ABTS, FRAP, and CUPRAC, respectively. On the other hand, flower extract displayed the highest antioxidant activity showing TE values of 431.12 ± 90.86, 15019.03 ± 698.72, and 920.33 ± 1.43 µmol/g for ABTS, FRAP, and CUPRAC, respectively. Herb extract also presented good results in both DPPH and ABTS (TE 303.90 ± 15.15 and 399.37 ± 21.50 µmol/g, respectively), while leaf extract had good results in DPPH (TE 319.78 ± 4.51 µmol/g) but was significantly less effective in ABTS (TE 215.01 ± 65.78 µmol/g) compared to flower extract. Both leaf and herb extracts showed significantly lower FRAP activity compared to the flower extract, but they were still more active than stem extract. According to Sepúlveda-Jiménez et al. results, leaf or flower extracts of *J. spicigera* had higher antioxidant activity than extract obtained from the stem and the order of free radical scavenging activity for methanolic extracts was leaf > flower > stem, whereas for aqueous extracts, this sequence was flower > leaf > stem [31]. Benabdallah et al. data showed that the methanolic extracts of *Mentha* L. species belonging to *Lamiaceae* family (also as *E. ciliata*) were rich in phenolic compounds and exhibited powerful antioxidant activity ranging from 7.5 µg/mL to 44.66 µg/mL of TE [26]. These results show that plants from the *Lamiaceae* family herbal materials are rich source of the phenolic compounds and their extracts have high antioxidant activity.

### 2.4. Identification and Quantification of Phenolic Compounds by High-Performance Liquid Chromatography (HPLC)

**Figure 4:**
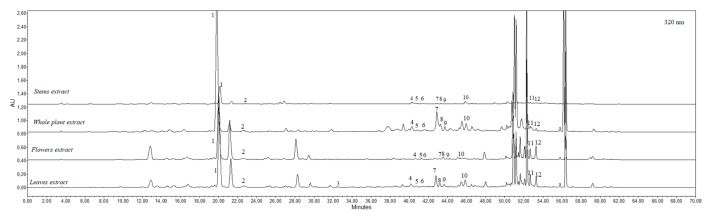
*E. ciliata* leaves, stems, flowers, and whole herb extracts phenolic profiles at 320 nm wavelenght: 1—chlorogenic acid, 2—caffeic acid, 3—*p*-coumaric acid, 4—rutin, 5—hyperoside, 6—luteolin-7-*O*-glucoside, 7—avicularin, 8—apigenin-7-*O*-glucoside, 9—quercitrin, 10—rosmarinic acid, 11—apigenin, 12—diosmetin.

Twelve phenolic compounds—rutin, hyperoside, quercitrin, avicularin, chlorogenic acid, rosmarinic acid, caffeic acid, *p*-coumaric acid, luteolin-7-*O*-glucoside, apigenin-7-*O*-glucoside, apigenin, and diosmetin—were identified by the HPLC analysis in the ethanolic extracts obtained from the leaf, stem, flower, and whole herb of *E. ciliata* (Figure 4). According to a recent study, the predominant compounds from *E. ciliata* ethanolic extract were luteolin, rosmarinic acid, and linarin [35]. In our previous study, 13 phenolic compounds were determined for the first time in *E. ciliata* dried herb extracts (quinic acid, neochlorogenic acid, chlorogenic acid, *p*-coumaric acid, vitexin, ferulic acid, luteolin-7-rutinoside, luteolin-7-glucoside, procyanidin B, apigenin-7-glucoside, naringenin, diosmetin, and chrysin) [24].

Amounts of individual phenolic compounds in *E. ciliata* ethanolic extracts are presented in Table 1.

The content of determined phenolic compounds of *E. ciliata* ethanolic extracts varies depending on plant part. The extract obtained from flower contained the significantly highest total amount of the quercetin glycosides—rutin, hyperoside, and avicularin—while stem extract had the lowest amounts of these compounds (Table 1). Whole plant extract presented significantly higher amounts of rutin and hyperoside (0.992 ± 0.079 and 0.126 ± 0.001 mg/g of DW, respectively) compared with leaves extract.

Rutin was the predominant compound in flowers, whole plant, and stems ethanolic extracts. The flower extract had the significant highest amount of rutin (2.286 ± 0.230 mg/g of DW) compared with leaf, stem, and whole plant extracts (*p* < 0.05). Avicularin was the second predominant compound in the ethanolic extracts obtained from flowers, whole plant, and leaves. Quercitrin was the minor component among all the quercetin glycosides quantified in all ethanolic extracts obtained from *E. ciliata* herbal materials.

All the quercetin glycosides identified and quantified in ethanolic extracts of *E. ciliata* samples can be ranked in the following ascending order by their content: In leaf extract—hyperoside < rutin < quercitrin < avicularin; stem extract—hyperoside < avicularin < quercitrin < rutin; whole herb extract –quercitrin< hyperoside < avicularin < rutin; flower extract—quercitrin < hyperoside < avicularin < rutin.

The highest total amount of phenolic acids identified and quantified by the HPLC method was found in the ethanolic extract obtained from the whole plant (Table 2).

The whole plant extract had the highest amount of chlorogenic acid (14910.91 ± 855.01 µg/g of DW) compared with leaf, stem, and flower extracts. The highest amount of rosmarinic acid (1.347 ± 0.443 mg/g DW) was obtained in extract produced from flower, and the lowest from leaf extract (0.115 ± 0.011 mg/g DW) (*p* < 0.05). Significant differences in the amounts of caffeic acid were obtained between all four ethanolic extracts (*p* < 0.05). The ethanolic extract of leaf contained the highest amount of caffeic acid (0.044 ± 0.0001 mg/g DW). In a recent study, the value of caffeic acid determined in *E. ciliata* whole plant ethanolic extract was 0.72 mg/g DW [36]. This amount of caffeic acid is higher than our study result, because after 60 min of sonification that extract was evaporated and freeze-dried (concentrated). Phenolic compound—*p*-coumaric acid was determined only in the extract obtained from *E. ciliata* leaf and it was 0.068 ± 0.003 mg/g of DW. According to our study results, chlorogenic acid and rutin can be chosen as phytochemical markers in the evaluation and control of quality parameters of *E. ciliata* herbal materials.

### 2.5. Analysis of the Antiradical Activity of E. ciliata Ethanolic Extracts Using HPLC-ABTS Post-Column Assay

**Figure 5:**
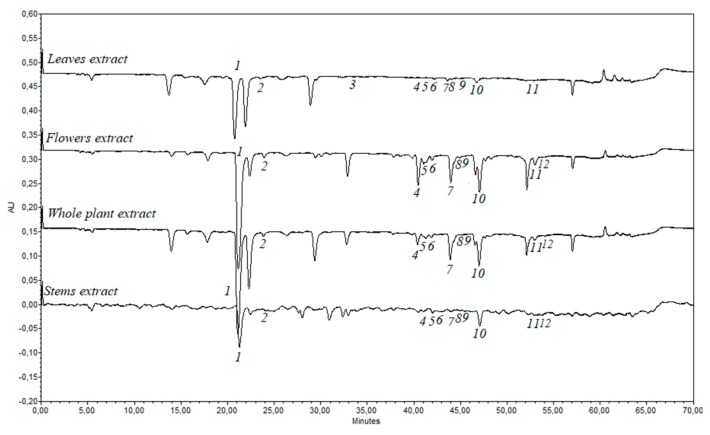
*E. ciliata* leaves, stems, flowers, and whole herb extracts ABTS post-column assay antiradical profile: 1—chlorogenic acid, 2—caffeic acid, 3—*p*-coumaric acid, 4—rutin, 5—hyperoside, 6—luteolin-7-*O*-glucoside, 7—avicularin, 8—apigenin-7-*O*-glucoside, 9—quercitrin; 10—rosmarinic acid, 11—apigenin, 12—diosmetin.

The human health benefits of plant-based raw materials for the accumulation of phenolic compounds are supported by a wealth of scientific data [37,38,39]. A link between the consumption of plant-based raw materials rich in these compounds and the incidence of oncological, cardiovascular, and neurodegenerative diseases has been established [40,41]. Identification of biologically active compounds with antioxidant activity is important. It allows scientifically substantiated use of medicinal herbal raw materials and their preparations for the prevention and treatment of oxidative stress disorders. It is expedient to evaluate the antioxidant activity of the plant raw material extracts provided by different anise, to identify analytical markers that could be used to evaluate the antioxidant activity and quality control of their medicinal products. The HPLC post-column method is significant because, unlike various UV-visible light spectrophotometry assays for the antioxidant activity of plant extracts, it allows the evaluation of the antioxidant activity of individual biologically active compounds from the extracts in vitro. The antiradical activity determination of the individual compounds of the *E. ciliata* plant raw material extracts was performed using an ABTS post-column assay [42]. Antioxidant activity profiles of *E. ciliata* herbal materials are presented in Figure 5.

HPLC-separated analytes in ABTS post-column assay react with ABTS radical cation, and bleaching is detected as negative peaks at 650 nm. Radical-scavenging activities of chlorogenic acid, caffeic acid, *p*-coumaric acid, rutin, hyperoside, luteolin-7-*O*-glucoside, avicularin, apigenin-7-*O*-glucoside, quercitrin, rosmarinic acid, apigenin, and diosmetin were determined in almost all antioxidant profiles. Table 3 represents quantitative values of antioxidant activity of compounds in ABTS post-column system. Total values of TE varies from 10.738 to 34.77 µmol/g DW in ABTS post-column assay. The highest TE values were obtained for ethanolic extract produced from flower, and the lowest for stem.

To the best of our knowledge, the antiradical activity of ethanolic extracts of different *E. ciliata* plant parts was analyzed for the first time. TE (µmol/g of DW) values of principal compounds and total of all quantitated compounds of *E. ciliata* leaf, stem, whole herb, and flower were assessed and presented in Table 3. The results demonstrate that *E. ciliata* flower and whole herb extracts (TE 34.20 ± 0.68 and 34.77 ± 0.68 µmol/g of ABTS, respectively) were the most antioxidant (*p* < 0.05) among all investigated extracts, but there was no significant difference between these two extracts (*p* > 0.05). The predominant radical scavengers in all *E. ciliata* ethanolic extracts were chlorogenic acid, rosmarinic acid, and avicularin. Antioxidant activity of chlorogenic acid comprises about 43% of total activity in flower extract and 33% in the whole herbal extract of *E. ciliata* and only about 21% of the total in stem extract in ABTS assay. In leaf extract, rosmarinic acid comprised only about 2% of total antioxidant activity, whereas in flower extract, this bioactive compound made about 13%; in the whole herb extract, about 9% of the radical scavenging activity. Avicularin antioxidant activity comprised about 6% of total activity in flower and whole herb extracts, and only about 0.7% of the total in stem extract. There are some unidentified compounds with high values of antioxidant activity, e.g., peaks at 13.93, 15.70, 17.59, 22.30, 26.54, 29.31, 50.13, 52.15, and 57.04 min). According to UV-Vis spectra, these unidentified compounds may be phenolic acids and/or flavonoids. HPLC-ABTS analysis may present antioxidative activity of the individual compounds, which consist of ethanolic extract composition. The *E. ciliata* extracts obtained from different plant parts have a potential antioxidant capacity, and the main antioxidative compounds of these extracts are chlorogenic acid, rosmarinic acid, and avicularin. These results may help for future studies to optimize extraction methods to reach the highest values of these antioxidative compounds to use them in food, pharmaceutical, or cosmetic fields.

### 2.6. Anti-Inflammatory Activity of the Ethanolic Extracts

Ethanolic extracts of leaf, stem, and flower parts of *E. ciliata* as well as of the whole herb were tested for anti-inflammatory activity in LPS-treated primary murine macrophage cell culture. Inflammation level was assessed by measuring the amounts of inflammatory mediators that are usually released by macrophages during bacterial infection: Cytokines TNF-α, IL-6, and PGE2 for determination the efficiency on cyclooxygenase-2 pathway.

**Figure 6:**
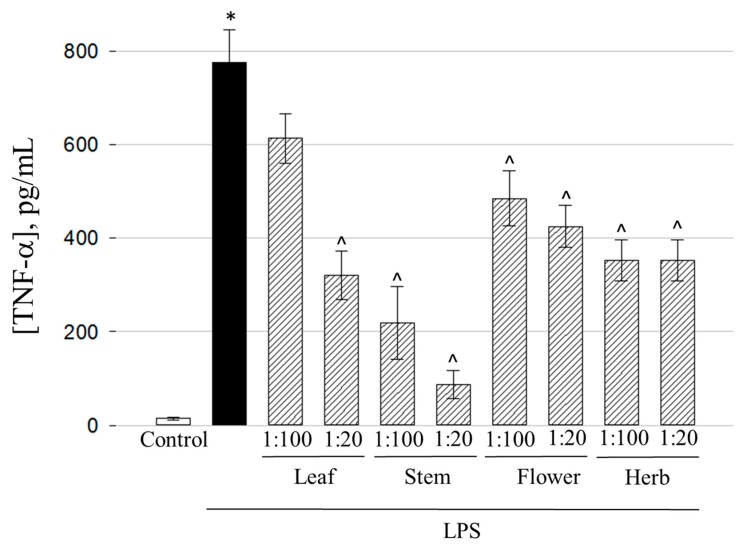
The effect of extracts obtained from different *E. ciliata* parts on LPS-induced TNF-α secretion. Results are presented as means ± SD. * significant difference compared to the Control, ^ compared to the LPS-only treatment (*p* < 0.05), one-way ANOVA with Tukey’s test.

**Figure 7:**
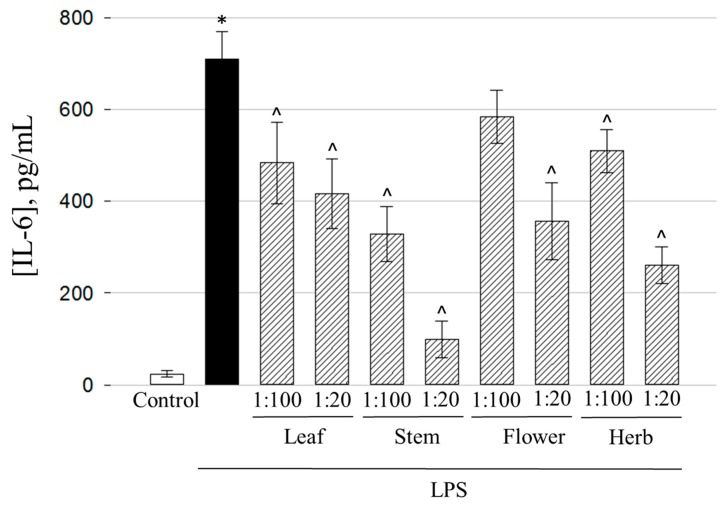
The effect of extracts obtained from different *E. ciliata* parts on LPS-induced IL-6 secretion. Results are presented as means ± SD. * Significant difference compared to the Control, ^ compared to the LPS-only treatment (*p* < 0.05), one-way ANOVA with Tukey’s test.

**Figure 8:**
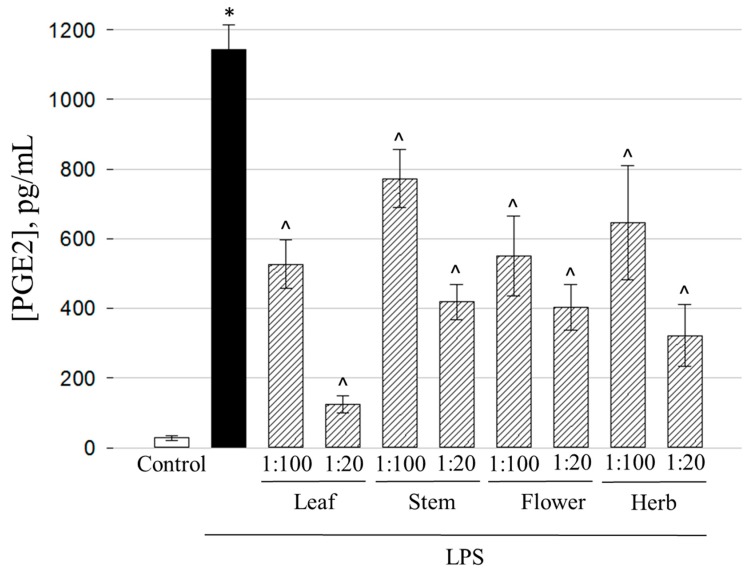
The effect of extracts obtained from different *E. ciliata* parts on LPS-induced Prostaglandin E2 secretion. Results are presented as means ± SD. * Significant difference compared to the Control, ^ compared to the LPS-only treatment (*p* < 0.05), one-way ANOVA with Tukey’s test.

After 24 h treatment with 1 µg/mL of LPS, the levels of inflammatory mediators secreted to the medium by cultured macrophages increased from below 30 pg/mL to 775 ± 70, 710 ± 60, and 1143 ± 70 pg/mL for TNF-α, IL-6, and PGE2, respectively (Figure 6, Figure 7 and Figure 8). When ethanolic extracts of *E. ciliata* were present together with the LPS treatment, the secretion of all three inflammatory mediators was significantly reduced with all tested preparations. The highest efficiency in decreasing TNF-α level was achieved by stem extract (Figure 6).

The concentrations of this cytokine dropped from 775 ± 70 to 87 ± 31 pg/mL in 1:20 dilution case reaching a level 9 times lower compared to LPS-treated samples. At 1:100 dilution, stem ethanolic extract decreased TNF-α level to 219 ± 78 pg/mL, which was 3.5 times lower compared to LPS-stimulated samples. There is a possibility of other non-phenolic compounds being the cause of the results exhibited by the stems extract, because this extract demonstrates the lowest amounts of bioactive compounds like phenolic acids and quercetin glycosides (Table 1 and Table 2). Both 1:100 and 1:20 flower extracts demonstrated moderate efficiency decreasing the cytokine level to 485 ± 59 and 425 ± 45 pg/mL, respectively. The predominant compounds in flower extract according to HPLC analysis results were rutin and chlorogenic acid (Table 1 and Table 2). The both chemicals are reported to be potent blockers of TNF-α-related inflammatory response [43,44], thus it is likely that rutin and chlorogenic acid might be responsible for prevention of release of this cytokine in our inflammation model. 1:100 leaf extract did not induce significant suppression of LPS-triggered TNF-α release, however at the higher concentration of 1:20, quite an efficient decrease to 321 ± 52 pg/mL was achieved.

Similar to the TNF-α case, the most efficient prevention of LPS-induced IL-6 release was demonstrated by 1:20 stem extract of *E. ciliata* (Figure 7).

The level of IL-6 dropped from 710±60 to 99±39 pg/mL, i.e., 7 times compared to LPS-treated samples. However, 1:100 stem extract had moderate efficiency and decreased IL-6 level only twice. A similar moderate effect on prevention of the release of the cytokine was found in 1:20 herb and 1:20 flower extract-treated samples. The highest values of phenolics, flavonoids, and antioxidant activity were obtained in flower extract (Figure 1, Figure 2 and Figure 3). Antioxidant activity is very tightly linked with anti-inflammatory efficiency and suppression of IL-6 release pathway, as demonstrated by Lowes et al. [45]. The researchers have found that secretion of IL-6 is prevented by antioxidants targeting mitochondrial oxidative phosphorylation system, thus similar activity might be exerted by the flower extract in our study. According to HPLC analysis results, flower extract had the highest amounts of rutin, hyperoside, and rosmarinic acid comparing with other extracts (Table 1 and Table 2). Ulrich-Merzenich et al. have investigated the anti-inflammatory activity of salicylate-based phytopharmaceuticals on human fibroblasts and discovered interaction of rutin with IL-6 release pathway [46]. Hyperoside was also identified as IL-6 and TNF-α suppressor in LPS-stimulated fibroblast cell culture and rheumatoid arthritis in vivo model [47]. Rosmarinic acid is well described for multiple anti-inflammatory actions including release of IL-6, TNF-α, and PGE2 [48,49]. This evidence suggests that anti-inflammatory activity of *E. ciliata* flower extract could be explained by the action of the three most abundant chemical substances of the extract. However, of note is that the highest efficiency against TNF-α and IL-6 release was discovered in the stem ethanolic extracts containing rutin and chlorogenic acid as predominant compounds compared with other phenolics. This finding indicates there might be other compounds with anti-inflammatory properties present in the extract such as triterpenes, sesquiterpenes, ketones, tannins, etc. The least effective were leaf and 1:100 herb extracts, and 1:100 flower extract did not significantly prevent the release of IL-6 induced by LPS.

Besides the cytokine signaling, another important proinflammatory pathway is related to the activity of cyclooxygenase-2 resulting in the formation of prostanoids including prostaglandins [50]. Thus, further in the study, we investigated how the extracts from different parts of the *E. ciliata* plant affect the secretion of the key mediator of this pathway PGE2. The experiments revealed that all the tested extracts have significant effect in preventing the release of the mediator in a dose-dependent manner (Figure 8).

1:20 leaf extract demonstrated the strongest capacity to suppress this inflammatory pathway. In the presence of the extract, the level of PGE2 in the medium of LPS-treated cells dropped from 1144 ± 70 to 125 ± 26 pg/mL, or 9 times. Flavonoids and other phenolic compounds are known to target cyclooxigenase-mediated inflammation [51,52,53], and one of the highest TPC, TFC, and antioxidant activity have been obtained in leaf extract (Figure 2, Figure 3 and Figure 4). According to HPLC analysis results, avicularin and quercitrin were the predominant compounds of quercetin glycosides in leaf extract (Table 2). Avicularin is demonstrated to block PGE2 release in LPS-treated murine macrophage cell line RAW 264.7 [54], however there is no evidence about anti-cyclooxigenase activity of quercitrin. According to phenolic acid content analysis, the leaf extract stood out among other extracts with the highest amount of caffeic acid (44.07 ± 0.14 µg/g of DW) (Table 2) (*p* < 0.05). This phenolic acid was demonstrated to block cyclooxygenase activity induced by UVB radiation [55], thus it might also be implicated in suppression of cyclooxygenase-mediated inflammatory pathway. PGE2 production can also be prevented by chlorogenic acids, as reported by Shi et al. [56]. Chlorogenic acid was the predominant phenolic acid in leaf extract (Table 2), and this also puts the compound on the list of potential candidates in prevention of cyclooxygenase-mediated inflammation signal. Also, potent prevention of PGE2 release was achieved by 1:20 whole herb, flower, and stem extracts. Concentration of PGE2 in the medium after treatment with LPS and the extracts were about 3 times lower than after treatment with LPS alone. Leaf and flower extracts at the concentration of 1:100 showed moderate PGE2 release blocking activity by decreasing the level of this mediator twice, whereas stem and whole herb extracts at this concentration prevented PGE2 release only by a quarter of the level caused by LPS alone.

Cell viability assessment by double nuclear staining revealed that none of the extracts at the concentrations used for the study were toxic to the cultured primary macrophages (Supplementary Figure S1). Also, there was no increase in cell death observed after the treatment with LPS (Supplementary Figure S1). This indicates that cytokine and PGE2 secretion changes observed in the study were caused by active redistribution of intracellular processes but not by change in the number of viable cells.

Summarizing, all the extracts investigated in the study prevented the LPS-induced release of inflammatory mediators in the dose-dependent manner, with an exception of herb extract effect on TNF-α release that was found the same at both 1:20 and 1:100 dilution. The most potent effect on release of pro-inflammatory cytokines TNF-α and IL-6 was revealed by 1:20 stem extract, whereas the best blocker of PGE2 release was 1:20 leaf extract. There was no significant effect on the level of inflammatory mediators in the cultures treated by the extracts alone without the presence of LPS (Supplementary Figure S1).

## 3. Materials and Methods

### 3.1. Plant Material

*E. ciliata* (Thunb.) Hyl. flower, leaf, stem, and whole plant were purchased from Zolynu namai, Vilnius, Lithuania. All the plant parts were separated manually and kept in dry, dark place for 5 days to dry. Dried herb was identified by Dr. Professor Nijole Savickiene, Medical Academy, Lithuanian University of Health Sciences. A voucher specimen (L 17710) was deposited at the Herbarium of the Department of Drug Technology and Social Pharmacy, Lithuanian University of Health Sciences. Dried flower, leaf, stem, and whole herb were separately grounded in a laboratory mill.

### 3.2. Chemicals

All the standards, reagents, and solvents used through experiments were of analytical grade. Apigenin, diosmetin, hyperoside, rutin, quercitrin, luteolin-7-*O*-glucoside, apigenin-7-*O*-glucoside, *p*-coumaric acid, caffeic acid, rosmarinic acid, and chlorogenic acid standards were purchased from Extrasynthese (Genay, France), and avicularin from Chromadex (Santa Ana, CA, USA). Acetic acid and acetonitrile were purchased from Sigma-Aldrich GmbH (Buchs, Switzerland), and ethanol from Vilniaus degtine, AB (Vilnius, Lithuania). Potassium persulfate, sodium acetate trihydrate, iron (III) chloride hexahydrate, and 2,4,6-tri(2-pyridyl)-*S*-triazine (TPTZ), 2,2-diphenyl-1-picrylhydrazyl (DPPH·) radical, and 2,2′-azino-bis(3-ethylbenzothiazoline-6-sulphonic acid) (ABTS) were obtained from Sigma-Aldrich (Steinheim, Germany) and Trolox from Fluka Chemika (Buchs, Switzerland). Folin-Ciocalteu reagent, gallic acid monohydrate, aluminum chloride hexahydrate, sodium carbonate, hexamethylenetetramine, copper chloride, and neocuproine were purchased from Sigma-Aldrich GmbH (Buchs, Switzerland). Deionized water used in HPLC and for the samples preparation produced by the Crystal E high-performance liquid chromatography (HPLC, Adrona SIA, Riga, Latvia) water purification system.

### 3.3. Preparation of E. ciliata Ethanolic Extracts

Powdered material of dried *E. ciliata* flower, leaf, stem, and whole plant (each for 1 g) was extracted with 20 mL of 70% (*v/v*) (1:20) ethanol in a round bottom flask by ultrasound-assisted extraction performed in an ultrasonic bath (Bandelin electronic GmbH & Co.KG, Berlin, Germany) at 25 °C for 30 min. The samples were centrifuged for 10 min at 4200 × g, followed by decantation of the supernatant. The extracts were filtered through paper and PVDF membrane filters (pore size 0.22 µm) prior to HPLC analysis.

### 3.4. Determination of Total Phenolic Content

All the spectrophotometric measurements were carried out with a UV/VIS 1800 Shimadzu spectrophotometer (Shimadzu, Japan). The total phenolic contents (TPC) in *E. ciliata* extracts were determined spectrophotometrically using the Folin–Ciocalteu method with some modifications [25]. Nearly 0.2 mL of dilute extract from each sample was mixed with 5 mL Folin-Ciocalteu reagent (diluted 10 times with distilled water). After 5 min, 4 mL of sodium carbonate solution (7.5%) was added and the mixture was kept in the dark place for 60 min. The absorbance of the resulting solution was measured at 765 nm. TPC was expressed in terms of milligrams of gallic acid equivalent (GAE) per gram of dry weight (mg GAE/g DW).

### 3.5. Determination of Total Flavonoid Content

The colorimetric aluminum chloride method with some modifications was used for quantification of the total flavonoid content (TFC) of the ethanolic extracts [57]. Briefly, 0.2 mL extract was mixed with 0.3 mL AlCl_3_ 10% aqueous solution. The absorbance of the reaction mixture was measured after 30 min incubation at 407 nm. The TFC was calculated from a calibration curve, and the result was expressed as mg rutin equivalent per g/DW.

### 3.6. Determination of Antioxidant Activity

#### 3.6.1. DPPH·Scavenging Assay

The DPPH· scavenging capacity was measured using the method proposed by Yim and Nam with slight modifications [58]. In this study, 10 μL of each ethanolic extract was mixed with 3 mL DPPH· solution. A decrease in absorbance was determined at a wavelength of 515 nm after keeping the samples for 30 min in the dark.

#### 3.6.2. ABTS·+ Decolorization Assay

An ABTS·+ decolorization assay was applied according to the methodology described by Yim and Nam [58]. A volume 10 μL of the ethanolic extract was mixed with 3 mL of ABTS·+ solution (absorbance 0.800 ± 0.02). A decrease in absorbance was measured at a wavelength of 734 nm after keeping the samples for 30 min in the dark.

#### 3.6.3. FRAP Assay

The ferric reducing antioxidant power (FRAP) assay was carried out as described by Benzie and Strain [59]. The working FRAP solution included TPTZ (0.01 M dissolved in 0.04 M HCl), FeCl_3_·6H_2_O (0.02 M in water), and acetate buffer (0.3 M, pH 3.6) at the ratio of 1:1:10. A volume of 3 mL of a freshly prepared FRAP reagent was mixed with 10 μL of the *E. ciliata* ethanolic extract. An increase in absorbance was recorded after 30 min incubation in the dark at a wavelength of 593 nm.

#### 3.6.4. CUPRAC Assay

The cupric ion reducing antioxidant capacity (CUPRAC) assay was applied according to the methodology described by Apak et al. [60]. A volume 10 μL of the ethanolic extract was mixed with 3 mL of CUPRAC solution. An increase in absorbance was recorded after 30 min incubation in the dark at a wavelength of 450 nm.

#### 3.6.5. Calculation of Antioxidant Activity of the *E. ciliata* Ethanolic Extracts

The antioxidant activity of extracts was calculated from Trolox calibration curve and expressed as μmol TE per gram of absolutely dry weight. TE was calculated according to the formula:

TE = c × V/m (μmol/g)(1)

where c is the concentration of Trolox established from the calibration curve (in μM); V is the volume of leaf extract (in mL); and m is the weight (precise) of grounded herbal powder (in g).

### 3.7. HPLC Analysis, ABTS Post-Column Assay

The chromatographic analysis was obtained according to Liaudanskas et al. [61]. The HPLC system applied consisted of Waters 2695 Alliance solvent manager (Waters, Milford, MA, USA) equipped with a Waters 996 photodiode array detector. Chromatographic separations were carried out by using a YMC-Pack ODS-A (5 μm, C_18_, 250 × 4.6 mm i.d.) column equipped with a YMC-Triart (5 μm, C_18_, 10 × 3.0 mm i.d.) precolumn (YMC Europe GmbH, Dinslaken, Germany). The column was operated at a constant temperature of 25 °C. The volume of the extract being investigated was 10 μL. The flow rate was 1 mL/min, and gradient elution was used. The mobile phase consisted of 2% (*v/v*) acetic acid in water (solvent A) and 100% (*v/v*) acetonitrile (solvent B). The following conditions of elution were applied: 0–30 min, 3%–15% B; 30–45 min, 15%–25% B; 45–50 min, 25%–50% B; and 50–55 min, 50%–95% B. The total duration of the analysis, including washing and reconditioning of the column, was 70 min. The confirmation of the chromatographic peak identity was achieved by comparing the retention times and spectral characteristics (λ = 200–600 nm) of the eluting peaks with those of reference compounds. For quantitative analysis, a calibration curve was obtained by injecting known concentrations (0.5–100 mg/mL) of different standard compounds.

HPLC post-column assay analysis was obtained according to Raudonis et al. [42]. HPLC post-column addition of ABTS solution was performed using continuously working Waters Reagent Manager (Milford, MA, USA) pump. The flow rate of the individual solutions was set at 0.5 mL/min. The mobile phase with separated analytes and ABTS solution flowed through a mixing tee to the reaction coil. The reaction coil was made of TFE (Teflon) tubing of the following size: 15 m × 0.3 mm i.d., 1.58 mm o.d., ~1 mL. The product chromatograms after ABTS post-column reaction were registered at 650 nm, using Waters 2487 dual λ absorbance (UV/Vis) detector (Milford, MA). Data received from experimental research were processed by Waters Empower software (Milford, MA).

ABTS solution was prepared freshly and kept at room temperature in darkness before HPLC post-column analysis.

The radical scavenging capacity was calculated and expressed as TE from Trolox calibration curves (ABTS R^2^ = 0.999). TE was calculated according to the formula:

(2) $$TE=\frac{S_{comp.}-b}{a}\times \frac{V_{s}}{m_{s}}$$

where *S_comp._* is the peak area of antioxidant active compound in the post-column chromatogram; *a* is slope; *b* is the y-intercept from Trolox calibration curve regressive equation; *V_s_* is the volume of herb raw material extract; and *m_s_* is the weight quantity of herb raw material.

### 3.8. Cell Culture and Treatments

**Figure 9:**
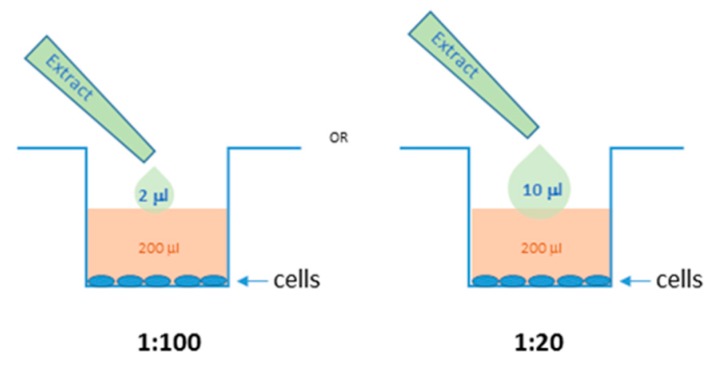
Ethanolic *E. ciliata* extracts dilution with cell medium for anti-inflammatory activity experiments.

All experimental procedures were performed according the Law of the Republic of Lithuanian Animal Welfare and Protection (License of the State Food and Veterinary Service for working with laboratory animals No. G2-80). The mice were maintained and handled at Lithuanian University of Health Sciences animal house in agreement with the ARRIVE guidelines. Peritoneal macrophages were isolated from 4-month-old Balb/c mice as described by Lu and Varley [62] with minor modifications. Briefly, after anesthesia and cervical dislocation, 5 mL of PBS solution was injected into the peritoneal cavity. The abdomen was gently massaged for 15–30 s, and the liquid from the peritoneal cavity collected with a syringe to a centrifuge tube and spun for 5 min at 300 *× g* (centrifuge Biosan BS-010212-AAA, Riga, Latvia). The pellet was resuspended in RPMI medium supplemented with 10% fetal calf serum (FCS), 20 ng/mL macrophage colony-stimulating factor (MCSF), and 100 IU/mL Penicillin-Streptomycin, and seeded into 96-well plates at a density of 470^5^ cells per well. The cells were kept in incubator (New Brunswick Galaxy 170S, Eppendorf, NY, USA) at 37 °C and 5% CO_2_. After 16 h, the cells were washed 3 times with the medium to remove non-macrophage cells. After another 24 h in the incubator (reaching about 75% of confluency), the cells were treated with 1 µg/mL lipopolysaccharide (LPS) from *E. coli* (Sigma Aldrich) with or without *E. ciliata* ethanolic extracts for 24 h. For anti-inflammatory experiments, the ethanolic extracts (ratio 1:20) were pipetted (2 and 10 µL) directly to the cell culture medium in the wells with cells (200 µL) (Figure 9). The final ratio of the extracts in cells medium were 1:20 and 1:100. After the treatment, cell culture media were collected for inflammatory mediator analysis and the cells were stained for viability evaluation.

### 3.9. Viability Staining

Cell viability was assessed by double nuclear fluorescent staining with Hoechst33342 (10 µg/mL) and propidium iodide (PI, 5 µg/mL), 5 min at 37 °C. PI-positive nuclei indicating lost nuclear membrane integrity were considered necrotic. Cells were visualized under fluorescent microscope OLYMPUS IX71S1F-3 (Olympus Corporation, Tokyo, Japan), counted in fluorescent micrographs by means of ImageJ freeware.

### 3.10. Inflammatory Mediator Detection

The levels of pro-inflammatory mediators in cell conditioned medium after treatments was detected by Enzyme-Linked Immuno Sorbent Assay (ELISA) kits for mouse tumor necrosis factor-α, or TNF-α (Thermo Fisher Scientific, Waltham, MA, USA), interleukin-6, or IL-6 (Thermo Fisher Scientific), and prostaglandin E2, or PGE2 (Abbexa Ltd.), according to protocols provided by the manufacturers. Optical density in the samples was measured in a MultiskanFC plate reader (Thermo Fisher Scientific). The data are presented as averages ± standard deviation.

### 3.11. Statistical Analysis

Statistical analysis was performed using one-way analysis of variance (ANOVA) followed by Tukey‘s test with the software SPSS Statistics 20.0 (IBM Corporation, NY, USA). The graphs were created by SigmaPlot v13. All quantitative data were done in triplicate, and the results are presented as means ± standard deviation. The value of *p* < 0.05 was taken as the level of significance.

## 4. Conclusions

The highest amounts of the main phenolic compounds—rutin, hyperoside, avicularin, and rosmarinic acid—were obtained in *E. ciliata* flower ethanolic extract, and quercitrin and chlorogenic acid in whole plant ethanolic extract. Flower, leaf, and whole plant ethanolic extracts showed the highest amounts of TPC, TFC, and antioxidant activity using DPPH and ABT assays. FRAP and CUPRAC assays indicated the highest antioxidant activity in the flower extract. As revealed by ABTS post-column analysis, the TE values of the flower extract was 1.7 times higher than leaf extract and 3 times higher than stem extract. Of all ethanolic extracts investigated, stem extract had the smallest amounts of quercetin glycosides, phenolic acids, TPC, TFC, and the lowest antioxidant activity using DPPH, ABTS, FRAP, and CUPRAC assays.

All investigated *E. ciliata* ethanolic extracts demonstrated anti-inflammatory activity. Stem and flower extracts most efficiently suppress TNF-α and IL-6-related pathways, and leaf extract is the most potent blocker of PGE2 secretion.

In general, this study shows that *E. ciliata* plant is a potential source of polyphenols and can be used as antioxidant and anti-inflammatory material. This study may serve as an incentive to continue the work already begun or to take new ones, investigating other biological effects and adapting the *E. ciliata* herbal material to various pharmaceutical forms.

## Figures and Tables

**Table 1:** Quantitative composition (mg/g of DW) of determined quercetin glycosides in *E. ciliata* ethanolic extracts.

Quercetin Glycosides				
Extracts	Rutin	Hyperoside	Quercitrin	Avicularin
*Leaves*	0.076 ± 0.002 ^a^	0.017 ± 0.001 ^a^	0.088 ± 0.004 ^a^	0.230 ± 0.009 ^a^
*Stems*	0.090 ± 0.001 ^a^	0.017 ± 0.001 ^a^	0.048 ± 0.002 ^b^	0.027 ± 0.001 ^c^
*Whole plant*	0.992 ± 0.079 ^b^	0.126 ± 0.001 ^b^	0.088 ± 0.002 ^a^	0.294 ± 0.034 ^a^
*Flowers*	2.286 ± 0.230 ^c^	0.233 ± 0.026 ^c^	0.075 ± 0.004 ^a^	0.481 ± 0.212 ^b^

Results are means ± SD (n = 3). Values with different lowercase letter(s) are significantly different in columns. (*p* < 0.05) as measured by Tukey’s test. DW—dry weight.

**Table 2:** Quantitative composition (mg/g of DW) of determined phenolic acids in *E. ciliata* ethanolic extracts.

Phenolic Acids				
Extracts	Chlorogenic Acid	Caffeic Acid	Rosmarinic Acid	*p*-Coumaric Acid
*Leaves*	10.477 ± 0.391 ^a^	0.044 ± 0.0001 ^a^	0.115 ± 0.011 ^a^	0.068 ± 0.003
*Stems*	2.748 ± 0.051 ^b^	0.035 ± 0.0008 ^b^	0.412 ± 0.017 ^ac^	ND
*Whole plant*	14.910 ± 0.855 ^c^	0.041 ± 0.0001 ^c^	0.959 ± 0.066 ^bc^	ND
*Flowers*	3.023 ± 0.051 ^bd^	0.038 ± 0.00002 ^d^	1.347 ± 0.443 ^d^	ND

Results are means ± SD (n = 3). Values with different lowercase letter(s) are significantly different in columns (*p* < 0.05) as measured by Tukey’s test. DW—dry weight; ND—not determined.

**Table 3:** Trolox equivalent (TE) values (µmol/g of DW) of *E. ciliata* ethanolic extracts in ABTS post-column assay.

Antioxidant Compound	RT (min)	Leaves Extract	Flowers Extract	Whole Herb Extract	Stems Extract
Unknown	13.93	2.12 ± 0.1	0.534 ± 0.03	2.24 ± 0.07	0.51 ± 0.02
Unknown	15.70	0.314 ± 0.01	0.322 ± 0.03	0.36 ± 0.01	0.17 ± 0.01
Unknown	17.59	1.153 ± 0.15	1.04 ± 0.05	1.46 ± 0.05	0.28 ± 0.02
Chlorogenic acid	21.66	5.423 ± 0.13	16.38 ± 0.08	12.43 ± 0.1	4.04 ± 0.06
Unknown	22.30	4.535 ± 0.09	1.851 ± 0.09	5.48 ± 0.05	0.57 ± 0.02
Caffeic acid	23.60	0.153 ± 0.03	0.396 ± 0.03	0.28 ± 0.01	0.56 ± 0.03
Unknown	26.54	0.394 ± 0.02	0.495 ± 0.01	0.56 ± 0.02	0.43 ± 0.02
Unknown	29.31	3.01 ± 0.08	0.359 ± 0.03	3.07 ± 0.06	0.52 ± 0.04
*p*-coumaric acid	32.41	0.142 ± 0.01	ND	ND	ND
Rutin	40.40	0.064 ± 0.01	2.257 ± 0.05	0.81 ± 0.03	0.21 ± 0.02
Hyperoside	41.03	0.061 ± 0.01	0.599 ± 0.03	0.22 ± 0.01	0.14 ± 0.01
Luteolin-7-*O*-glucoside	41.80	0.132 ± 0.02	0.287 ± 0.03	0.20 ± 0.01	0.33 ± 0.03
Avicularin	43.95	0.268 ± 0.02	2.519 ± 0.06	1.74 ± 0.06	0.16 ± 0.01
Apigenin-7-*O*-glucoside	44.90	0.083 ± 0.01	0.358 ± 0.03	0.03 ± 0.001	0.06 ± 0.01
Quercitrin	45.25	0.13 ± 0.01	0.11 ± 0.01	0.12 ± 0.02	0.048 ± 0.0
Rosmarinic acid	47.02	0.501 ± 0.02	3.806 ± 0.02	3.32 ± 0.08	1.23 ± 0.06
Unknown	50.13	0.028 ± 0.01	0.061 ± 0.01	0.1 ± 0.01	0.62 ± 0.02
Unknown	52.15	0.07 ± 0.01	2.059 ± 0.06	1.38 ± 0.06	0.19 ± 0.02
Apigenin	53.70	0.045 ± 0.01	0.072 ± 0.01	ND	0.32 ± 0.03
Diosmetin	54.15	ND	0.038 ± 0.01	0.07 ± 0.01	0.11 ± 0.02
Unknown	57.04	0.74 ± 0.03	0.654 ± 0.01	0.9 ± 0.02	0.24 ± 0.01
*Total of all quantitated compounds*		19.37 ± 0.78 ^a^	34.20 ± 0.68 ^b^	34.77 ± 0.68 ^b^	10.74 ± 0.47 ^c^

ND—not determined, Values with different lowercase letter(s) are significantly different in columns (*p* < 0.05) as measured by Tukey’s test.

## Supplementary Materials

The following are available online: Figure S1: Representative images of macrophage viability assessment by double nuclear fluorescent staining. (a) Control, (b) treated with 1:20 E. ciliata Leaf extract, (c) 1:20 Stem extract, (d) 1:20 Flower extract, (e) 1:20 Herb extract, (f) 1 µg/mL LPS, (g) LPS and 1:20 Leaf extract, (h) LPS and 1:20 Stem extract, (i) LPS and 1:20 Flower extract, (j) LPS and Herb extract. All nuclei are Hoechst33342-only positive and are visible as blue, and necrotic nuclei are both Hoechst3342 and propidium iodide-positive and are visible as red or purple. The percentage of necrotic nuclei in all samples investigated were in the range between 95 ± 4 and 99 ± 3 (average of 5 microscopic fields of 3 different wells was counted in 3 separate experiments for each sample). Based on this result, it was assumed that the treatments did not affect cell viability.
