# Supplementary material for: Elsholtzia ciliata (Thunb.) Hyl. Extracts from Different Plant Parts: Phenolic Composition, Antioxidant, and Anti-Inflammatory Activities
Source: Molecules. 2020 Mar 5;25(5):1153. doi: 10.3390/molecules25051153 (PMC7179165; doi:10.3390/molecules25051153)
Supplement: Supplementary file 1 [file molecules-25-01153-s001.pdf]

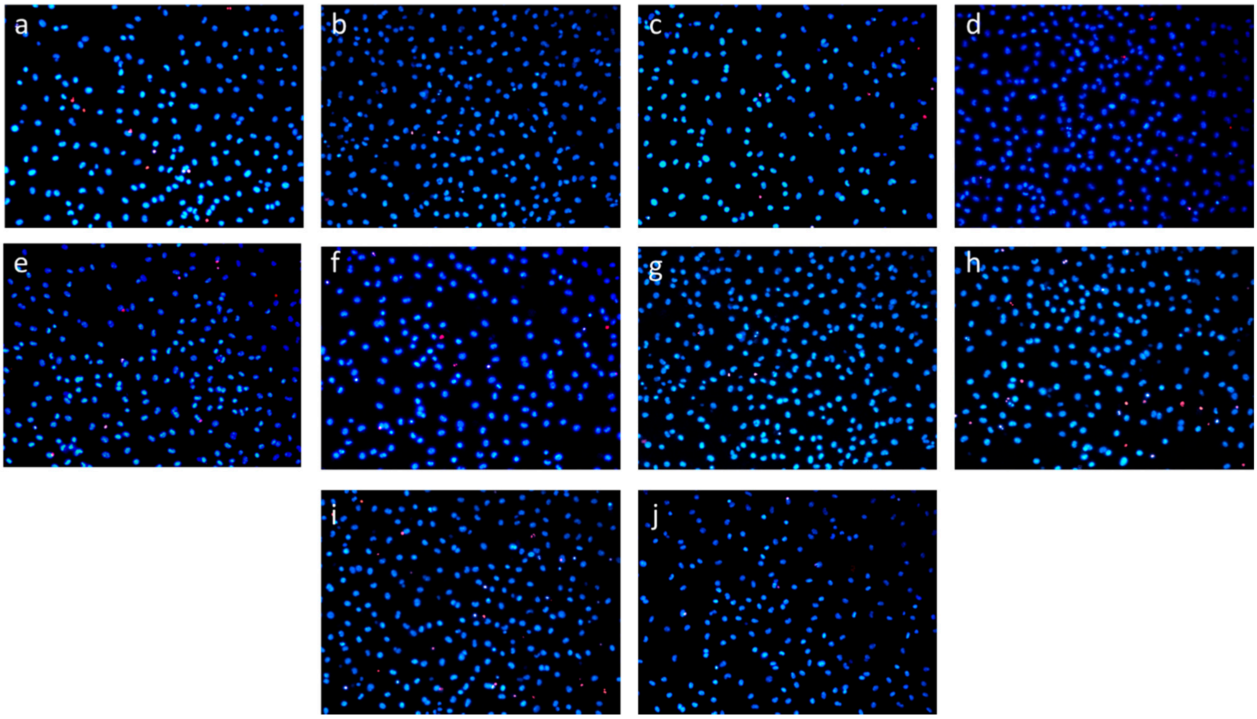

Figure S1. Representative images of macrophage viability assessment by double nuclear fluorescent staining. a) – control, b) – treated with 1:20 *E. ciliata* Leaf extract, c) – 1:20 Stem extract, d) – 1:20 Flower extract, e) – 1:20 Herb extract, f) - 1 µg/mL LPS, g – LPS and 1:20 Leaf extract, h) - LPS and 1:20 Stem extract, i) - LPS and 1:20 Flower extract, j) - LPS and Herb extract. All nuclei are Hoechst33342-only positive and are visible as blue, and necrotic nuclei are both Hoechst3342 and propidium iodide-positive and are visible as red or purple. The percentage of necrotic nuclei in all samples investigated were in the range between 95±4 and 99±3 (average of 5 microscopic fields of 3 different wells was counted in 3 separate experiments for each sample). Based on this result, it was assumed that the treatments did not affect cell viability.
